# Testing the potential of a virtual reality neurorehabilitation system during performance of observation, imagery and imitation of motor actions recorded by wireless functional near-infrared spectroscopy (fNIRS)

**DOI:** 10.1186/1743-0003-7-57

**Published:** 2010-12-02

**Authors:** Lisa Holper, Thomas Muehlemann, Felix Scholkmann, Kynan Eng, Daniel Kiper, Martin Wolf

**Affiliations:** 1 Biomedical Optics Research Laboratory (BORL), Division of Neonatology, Department of Obstetrics and Gynecology, University Hospital Zurich, Frauenklinikstrasse 10, 8091 Zurich, Switzerland; 2 Institute of Neuroinformatics (INI), University of Zurich and ETH Zurich, Winterthurerstrasse 190, 8057 Zurich, Switzerland; 3 Molecular Imaging and Functional Pharmacology, Institute for Biomedical Engineering, ETH and University of Zurich, Wolfgang-Pauli-Strasse 27, 8093 Zurich, Switzerland

## Abstract

**Background:**

Several neurorehabilitation strategies have been introduced over the last decade based on the so-called simulation hypothesis. This hypothesis states that a neural network located in primary and secondary motor areas is activated not only during overt motor execution, but also during observation or imagery of the same motor action. Based on this hypothesis, we investigated the combination of a virtual reality (VR) based neurorehabilitation system together with a wireless functional near infrared spectroscopy (fNIRS) instrument. This combination is particularly appealing from a rehabilitation perspective as it may allow minimally constrained monitoring during neurorehabilitative training.

**Methods:**

fNIRS was applied over F3 of healthy subjects during task performance in a virtual reality (VR) environment: 1) 'unilateral' group (N = 15), contralateral recording during *observation*, *motor imagery*, *observation & motor imagery*, and *imitation *of a grasping task performed by a virtual limb (first-person perspective view) using the right hand; 2) 'bilateral' group (N = 8), bilateral recording during *observation *and *imitation *of the same task using the right and left hand alternately.

**Results:**

In the unilateral group, significant within-condition oxy-hemoglobin concentration Δ[O_2_Hb] changes (mean ± SD μmol/l) were found for *motor imagery *(0.0868 ± 0.5201 μmol/l) and *imitation *(0.1715 ± 0.4567 μmol/l). In addition, the bilateral group showed a significant within-condition Δ[O_2_Hb] change for *observation *(0.0924 ± 0.3369 μmol/l) as well as between-conditions with lower Δ[O_2_Hb] amplitudes during *observation *compared to *imitation*, especially in the ipsilateral hemisphere (p < 0.001). Further, in the bilateral group, *imitation *using the non-dominant (left) hand resulted in larger Δ[O_2_Hb] changes in both the ipsi- and contralateral hemispheres as compared to using the dominant (right) hand.

**Conclusions:**

This study shows that our combined VR-fNIRS based neurorehabilitation system can activate the action-observation system as described by the simulation hypothesis during performance of observation, motor imagery and imitation of hand actions elicited by a VR environment. Further, in accordance with previous studies, the findings of this study revealed that both inter-subject variability and handedness need to be taken into account when recording in untrained subjects. These findings are of relevance for demonstrating the potential of the VR-fNIRS instrument in neurofeedback applications.

## Introduction

### Neurorehabilitation based on the simulation hypothesis

Over the last decades, promising strategies in neurorehabilitation, e.g. following cerebral stroke [1-3], have been introduced based on the so-called simulation hypothesis [4,5]. The hypothesis suggests that the neural networks of a action-observation system located in the primary motor cortex (M1) and secondary motor areas, such as premotor cortex (PMC), supplementary motor area (SMA) and the parietal cortices, are not only activated during overt motor execution, but also during observation or imagery of the same motor action [6]. These networks are activated when individuals learn motor actions via execution (as in traditional motor learning), imitation, observation (as in observational learning) and motor imagery. Activation of these brain areas following observation or motor imagery may thereby facilitate subsequent movement execution by directly matching the observed or imagined action onto the internal simulation of that action [7]. It is therefore believed that this multi-sensory action-observation system enables individuals to (re)learn impaired motor functions through the activation of these internal action-related representations [8].

We have integrated this knowledge in a novel neurorehabilitative treatment system, based on motor and imagery performance in a virtual reality (VR) environment [9]: the system consists of a VR environment containing virtual representations of the patient's own arms and hands, which are displayed on a large screen and controlled by the patient wearing arm position trackers and data gloves. To activate the action-observation system, patients can train impaired upper limb function by playing interactive games in which they have to perform or imagine specific upper limb movements to interact with the VR environment. By adjustably mapping the movements of both the paretic and healthy limb onto the virtual limbs, the system offers individual training of upper limb motor function even in patients with little arm or hand movement ability.

### Functional near-infrared spectroscopy

To monitor the VR system's effects on brain activation, we chose functional near-infrared spectroscopy (fNIRS). fNIRS is a non-invasive technique based on neurovascular coupling, which exploits the effect of metabolic activity due to neural processing on the oxygenation of cerebral tissue. Utilizing this tight coupling between neuronal activity and localized cerebral blood flow, fNIRS measures hemodynamic changes associated with cortical activation [10]. Optical NIR technology has been shown to be a reliable tool for functional neuroimaging of the human brain [11]. Although NIR technologies feature lower spatial resolution and are only able to image cortical tissue while not providing deeper tissue interrogation as compared to traditional neuroimaging methods such as functional magnetic resonance imaging (fMRI), they offer the advantage of portable systems and, in theory, insensitivity to electromagnetic fields and ferromagnetic materials. In this study a novel miniaturized wireless fNIRS instrument was used [12]. This wireless and portable NIRS technology does not require the subject's body or head to be restrained, and therefore represents an optimal brain monitoring tool for our purpose to record from subjects performing movements in a VR environment. It is thought that this wireless fNIRS technology could overcome some of the limitations inherent to traditional neuroimaging methods.

While the action-observation system described above has been widely investigated using traditional neuroimaging methods [13-15], so far there are only a few studies using NIRS based techniques [16-19]. Further studies have shown fNIRS to be a reliable tool to measure brain oxygenation related to motor imagery performance [20-27], confirming the well-known cortical areas located in primary and secondary motor areas.

The focus of the present study was to obtain evidence of the VR system's efficacy in neurorehabilitation by evaluating its effects on brain activation. In particular, we aimed 1) to provide evidence, that our VR system is able to elicit the action-observation system and 2) to draw conclusions for the system's further application in neurorehabilitative treatment. We hypothesized that the observation, imagery and imitation of a hand motor task in an interactive VR environment enhances the related cortical oxygenation changes of the action-observation system as measured by fNIRS. The long-term aim is to implement the data obtained in the development of a VR-fNIRS based brain computer interfaces (BCIs). Such a VR-fNIRS based BCI could enhance patients' motivation by providing real-time neurofeedback thereby allowing therapists to record pre-post treatment progress assessing training-induced oxygenation changes.

## Materials and methods

### Subjects

Right-handed subjects were recruited via advertisements at the University of Zurich and ETH Zurich. Exclusion criteria were any history of visual, neurological or psychiatric disorders or any current medication. All subjects gave informed consent. All subjects had normal or corrected-to-normal vision. The study was approved by the ethics committee of the Canton of Zurich and was in accordance with the latest version of the Helsinki declaration.

### Experimental protocol

Prior to recording, subjects completed the Edinburgh Handedness Inventory (EHI) [28] assessing hand dominance to exclude left-handed subjects. The right-handed subjects were assigned to one of two groups: either to the 'unilateral' group (N = 15) or to the 'bilateral' group (N = 8). Each subject in either group participated in one experimental session. However, bilateral wireless NIRS measurements are more demanding with respect to the instrumentation: two devices are needed instead of one and they must be time-synchronized.

All experiments were conducted in a quiet room. Subjects sat in front of a custom made VR table-system with a computer screen (94 cm diagonal) to display the VR environment [9]. The subjects were asked to place their hands on the table with the palms facing downwards, and faced the monitor at a distance of approximately 70 cm. The image on the monitor showed a virtual arm in the same orientation and relative position as the real arms, resting on a flat surface representing the table. The close correspondence between the virtual and real arms in terms of position and relative (first-person) orientation was designed to optimally stimulate the patient to imagine the virtual arms as their own during the experimental session.

#### Unilateral group

**Figure 1:**
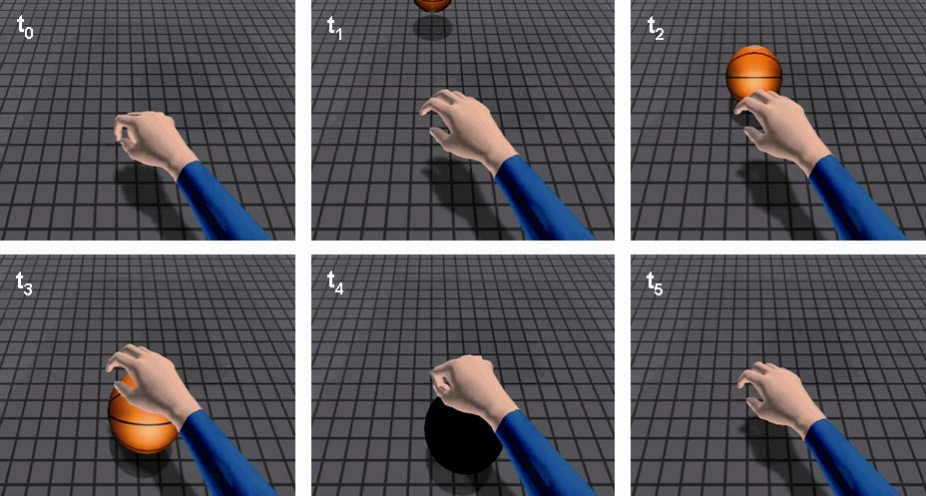
**Ball catching task (13 actions in 20 s) as shown in the VR video (from top left to bottom right)**.

In the subject group 'unilateral', fNIRS was recorded over the left hemisphere while the subject performed the VR tasks under four conditions:

▪ 'Observation (O)': subjects passively watched a VR video which displayed a right upper limb with the hand repeatedly grasping an incoming ball (13 actions, approx. 0.86 Hz) (Figure 1).

▪ 'Observation & motor imagery (O&MI)': same as condition O, except that subjects were asked to imagine that the virtual arm was their own.

▪ 'Motor imagery (MI)': same as condition O&MI, but without visual input - subjects had to imagine performing the action.

▪ 'Imitation (IM)': subjects imitated the hand movements performed in the VR task by the virtual arm while watching the VR video.

**Figure 2:**
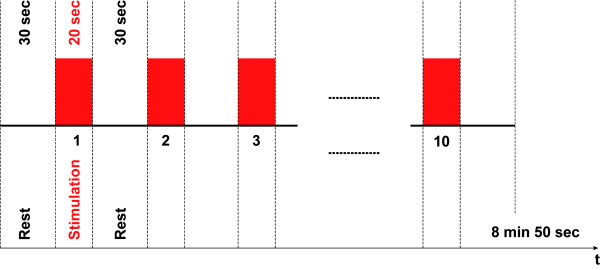
**Experimental block design. Each condition consisted of an initial rest period of 30 s, followed by 10 stimulation periods (20 s) alternated with rest periods (30 s)**. Each condition lasted 530 s (8 min 50 s); the total duration of the experiment was approx. 35 min per subject.

The session began with a practice trial (approx. 5 min) to allow subjects to become familiar with the tasks. After the practice trial, all subjects first performed condition O followed by a randomized presentation of conditions O&MI, MI and IM (Easy Randomizer, Version 4.1. [29]). Subjects were reminded to perform the executed or imagined movements with the same frequency as shown in the video (approx. 0.86 Hz). Each condition lasted 530 s (8 min 50 s) consisting of 10 trials each comprising an initial rest period (30 s), followed by 10 stimulation periods (20 s) alternated with rest periods (30 s) (Figure 2). The total number of trials per subject was 40; the total duration of the experiment was approx. 35 min per subject. We chose these irregular periodic alternations of 20 s stimulation and 30 s rest periods to avoid the induction of synchronization of the sequence of the motor stimulation/rest periods in the motor stimulation protocol with systemic rhythms such as heartbeat, respiration and heart rate fluctuations.

#### Bilateral group

The subject group 'bilateral' had the same VR task as the group 'unilateral', but was recorded bilaterally. This group was included to test for a lateralized distribution of oxygenation patterns for the ipsi- and contralateral side, as seen in related studies [30-33]. We hypothesized that, on the one side, the hemisphere contralateral to the hand performing the task would show larger [O_2_Hb] changes as compared to the ipsilateral hemisphere. The detection of larger [O_2_Hb] changes over the hemisphere contralateral would provide evidence that we were indeed recording from the correct position, i.e. covering motor-related cortical areas. Conditions O and MI were chosen as we assumed that these conditions would elicit the smallest oxygenation changes, both unilaterally and bilaterally. Therefore conditions O&MI and MI were dropped as we assumed that these conditions would follow a similar pattern to the other conditions.

▪ 'Observation right (O_R)': Same as condition O in the unilateral group.

▪ 'Observation left (O_L)': Same as condition O_R, except that a left hand was shown in the VR video.

▪ 'Imitation right (IM_R)': Same as condition IM in the unilateral group.

▪ 'Imitation left (IM_L)': Same as condition IM_R, except that a left hand was shown in the VR video and subjects were asked to imitate the movement with their left hand.

After the practice trial, all subjects performed condition O_R or O_L first, which was randomly assigned, followed by condition IM_R or IM_L, which was also randomized (Easy Randomizer, Version 4.1. by [29]). The procedure and timing were the same for both the 'unilateral' and the 'bilateral' groups.

### NIRS instrumentation

**Figure 3:**
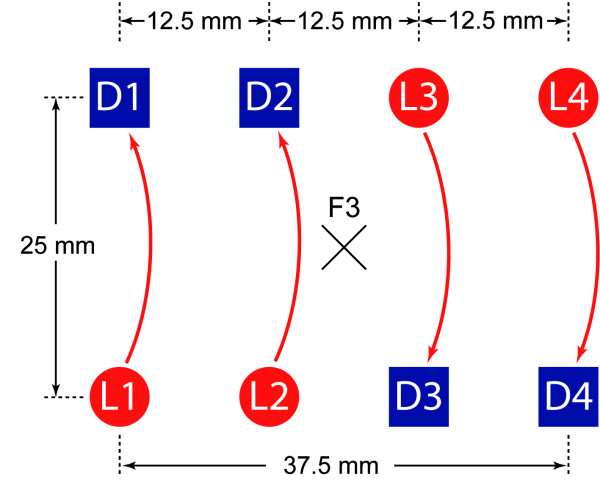
**Top-view: schematic of light sources (L1, L2, L3, and L4) and detectors (D1, D2, D3, and D4) on the sensor**. The center of the sensor was positioned over position F3 according to the 10-20 system. Four channels were considered for analysis. D1-L1 were in cranial direction, D4-L4 were in caudal direction.

The novel miniaturized continuous wave wireless fNIRS sensor has been previously described in detail [12]. The optical and electronic components are mounted onto a four-layer rigid-flexible printed circuit board (PCB) which, in combination with a highly flexible casing made of medical grade silicone, enables the sensor to be aligned to curved body surfaces such as the head. The size of the device is 92 × 40 × 22 mm and weighs 40 g. The optical system comprises four light sources at two different wavelengths (760 nm and 870 nm) and four detectors (PIN silicon photodiodes). The distance between light sources and detectors is 25 mm, four light source-detector pairs are linearly arranged every 12.5 mm and thus cover an area of 37.5 × 25 mm (Figure 3). Each light source consists of two pairs of serially connected light emitting diodes (LED) is driven using current control and is time multiplexed with an on-time of 120 μs per sample and a forward voltage of 4 V per diode. Although LEDs have a broader emission spectrum than lasers, they have several advantages: they can be applied directly on the body surface without need for lenses or fibers and they are inexpensive. Furthermore, they are harmless for the eye, which is an important advantage in a clinical environment. The power is provided by a rechargeable battery, which allows continuous data acquisition for 180 minutes at full light emission power. The light intensity is sampled at 100 Hz and the resulting data are transmitted wirelessly to the host computer by Bluetooth. The operating range of the sensor is about 5 m. The wireless sensor has been found to be capable of detecting both localized changes [O_2_Hb] and [HHb] in the adult brain and oxygenation changes of muscular tissue [12,34].

For fNIRS recording, the sensor(s) was(were) placed either contralateral (unilateral group) or bilaterally (bilateral group) on the subject's head presumably covering F3 according to the international 10-20 system [35]. With the compact sensor of 37.5 mm length and 25 mm width, we assumed that we covered secondary motor areas [36]. Hairs under the sensor(s) were carefully brushed away before fixation; shaving was not required. The sensor was fixed on the subject's head using medical-grade, disposable, self-adhesive bandages (Derma Plast CoFix 40 mm, IVF Hartmann, Neuhausen, Switzerland).

For final data processing, by measuring intensity of NIR light after its transmission trough tissue, it is possible to determine changes over time in the concentration of oxy-hemoglobin (O_2_Hb) and deoxy-hemoglobin (HHb), which represent the dominant light absorbers for living tissue in the NIR spectral band. By applying the modified Beer-Lambert law (MBBL), the concentration for O_2_Hb and HHb ([O_2_Hb], [HHb]) were computed from the measured absorption changes [37,38].

A MATLAB^® ^(Version 2008a) program was applied to pre-process the raw light intensity values and to compute [O_2_Hb] and [HHb] changes. The measurement files that were acquired during the fNIRS experiment contain the intensity signals of the NIR light, sampled at 100 Hz for all combinations of light-sources, wavelengths and detectors, as well as the intensity of the ambient light. The program subtracts the ambient light intensities from the NIRS measurement values before low-pass filtering (7^th ^order Chebyshew with 20 dB attenuation at 5 Hz) and decimating the signals to a sampling rate of 10 Hz. Consecutively, the MBLL is used to compute the changes of [O_2_Hb] and [HHb] applying differential path lengths factors (DPF) of 6.75 for the 760 nm and 6.50 for the 870 nm light-sources [39]. The [O_2_Hb] and [HHb] signals acquired with the wireless NIRS signal characteristically drift slightly over time, which can mostly be attributed to thermal effects. Therefore, data was recorded only two minutes after starting the fNIRS sensor, allowing the setup to reach thermal equilibrium. The remaining signal drift [12] was highly linear as assessed by visual inspection and thus their linear least squares approximation was subtracted from [O_2_Hb] and [HHb] for drift elimination.

## Data Analysis

Descriptive analysis was calculated for all median signal amplitudes (μmol/l ± SD). Each source-detector combination (channel) and each condition was averaged to attempt to provide a detectable signal. The criterion for a detectable signal was the relative value between stimulation and baseline, i.e. increase in [O_2_Hb] and decrease in [HHb]. At this point those channels that did not show task related oxygenation changes were excluded from further analysis, since it was assumed that those channels did not cover the activated cerebral region at all. For the same reason, subjects that did not display statistically significant changes of the [O_2_Hb] median for the condition IM (control condition) were excluded as well.

All data were positively tested for Gaussian distribution using the Kolmogorov-Smirnov test. Consecutively, dependant variables for further statistical analysis were derived from the non-excluded [O_2_Hb] and [HHb] datasets. Specifically, the median of the last 10 s of the stimulation periods ([HHb]_stim_, [O_2_Hb]_stim_, stimulation amplitudes) and the median of the last 10 s of the rest periods ([HHb]_rest_, [O_2_Hb]_rest_, baselines) were tested in the analysis. The median was chosen instead of the mean as it is more robust to outliners that may have statistically unbalanced the analysis in our relatively small subject sample. The statistical significance of the intra-condition differences between ([HHb]_rest_, [O_2_Hb]_rest_) and ([HHb]_stim_, [O_2_Hb]_stim_), later referred to as Δ[HHb] and Δ[O_2_Hb], was analyzed using the paired t-test.

The statistical significance of inter-conditional differences of [O_2_Hb]_stim _and [HHb]_stim _as well as for [HHb]_rest _and [O_2_Hb]_rest _were first assessed across all conditions. Then, if a significant difference was found, it was followed by a pair wise comparisons for all possible condition pairs using one-way ANOVA; the alpha-value for significance was set to ≤ 0.05 and the Bonferroni correction was applied to eliminate the problem of multiple comparisons.

## Results

### Behavioral data

23 healthy subjects were included in the analysis (15 unilateral group, 8 bilateral group, 9 males, mean age 26 years, range 22 - 33 years). Five subjects (2 in unilateral group; 3 in bilateral group) were excluded from analysis due to a missing signal in the IM condition. All subjects were right-handed according to the EHI with a mean LQ of 81.9 (range 73 - 100) and a mean deciles level of 6.1 (range 3 - 10).

### fNIRS measurements

#### Unilateral group

The mean Δ[O_2_Hb] (Table 1) was largest in the IM condition, followed by MI, O, and O&MI. Mean Δ[HHb] was largest in condition MI, followed by IM, O&MI, and O. The data showed a higher degree of inter-subject variability observed for Δ[O_2_Hb] compared to Δ[HHb] as calculated by the standard deviation (SD) of the oxygenation changes.

**Table 1:** Unilateral group.

Unilateral group [N = 15]	Observation	Motor imagery	Observation & motor imagery	Imitation
left hemisphere (contralateral) (μmol/l ± SD)				

Mean Δ[O_2_Hb]	0.0692 ± 0.4510	0.0868 ± 0.5201	0.0446 ± 0.5741	0.1715 ± 0.4567

Mean Δ[HHb]	-0.0052 ± 0.1247	0.0356 ± 0.2043	-0.0089 ± 0.2391	0.0212 ± 0.1685

				

T-test, CI 95%				

[O_2_Hb] p = value	p = 0.154	p = 0.049*	p = 0.333	p < 0.001*

[HHb] p-value	p = 0.161	p = 0.061	p = 0.760	p = 0.323

				

ANOVA, post-hoc-tests, Bonferroni 0.05		[HHb] p-value	[O_2_Hb] p = value	

	O - MI	p = 0.387	p = 1.000	

	O - O&MI	p = 1.000	p = 1.000	

	O - IM	p = 1.000	p = 0.509	

	MI - O&MI	p = 0.265	p = 1.000	

	MI - IM	p = 1.000	p = 0.934	

	O&MI - IM	p = 1.000	p = 0.194	

(Top) Mean signal amplitudes (μmol/l ± SD) of channels with significant concentration changes. Separate calculations for increases in [O2Hb], decreases in [HHb] in response to the four conditions for each group. Numbers were rounded to four decimal places. (Middle) Intra-condition statistical significance of the mean changes between [O2Hb]rest and [O2Hb]stim and [HHb]rest and [HHb]stim using the paired t-test; confidence interval (CI) = 95%. (Bottom) Inter-condition statistical significance of mean changes of Δ[O2Hb] and Δ[HHb] between the four conditions using ANOVA. Shown are post-hoc tests (with Bonferroni correction); significant values (p ≤ 0.05) are highlighted by * (observation = O, motor imagery = MI, observation & motor imagery = O & MI, imitation = IM)

**Figure 4:**
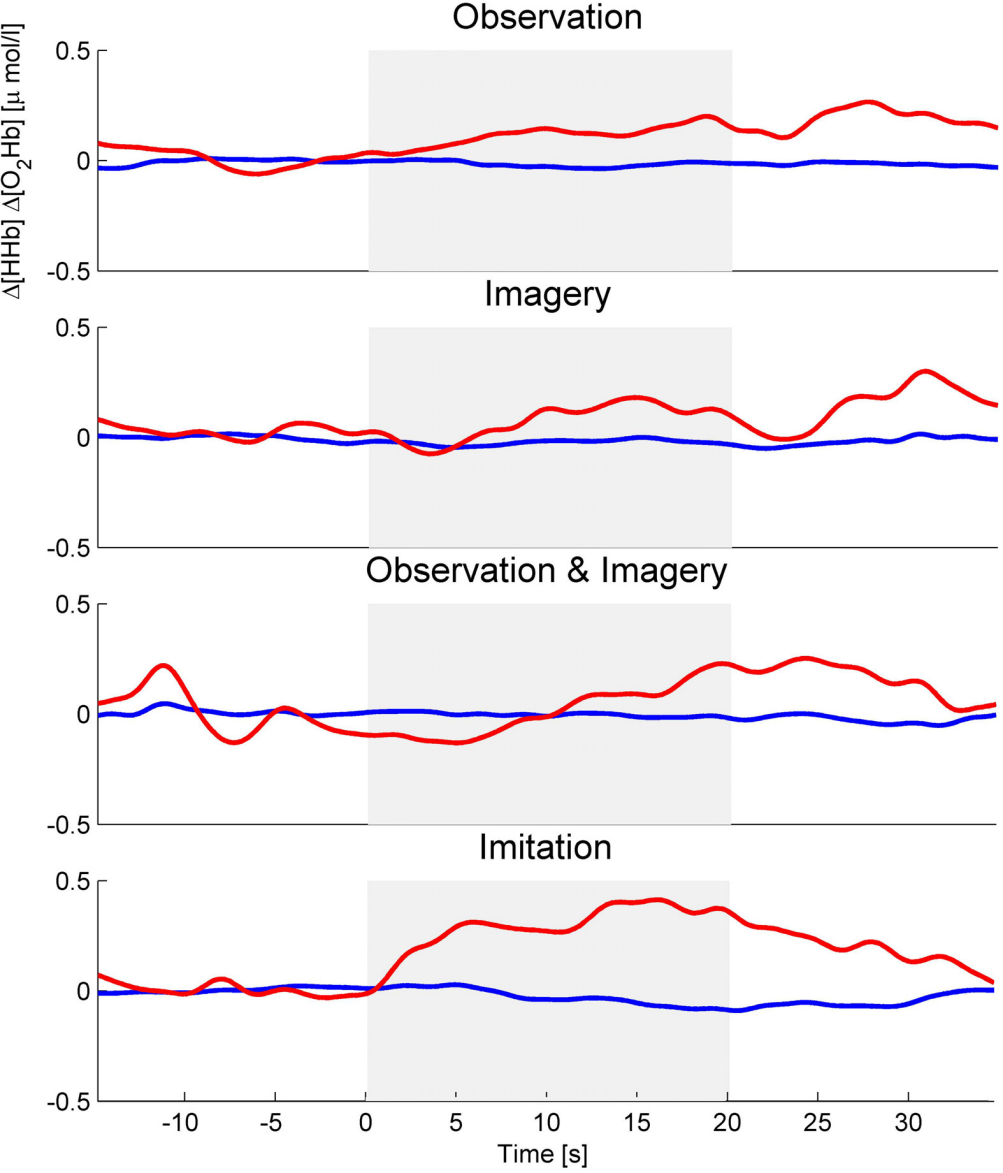
**Example of a sample subject of the oxygenation changes Δ[O_2_Hb] and Δ[HHb] (μmol/l) from rest (30 s) to stimulation (20 s) period in each of the four conditions Observation (O), Imagery (MI), Observation & Imagery (O&MI) and Imitation (IM)**. Stimulation on- and offset is indicated by the dotted lines.

Intra-condition analysis of the median changes between [O_2_Hb]_rest _and [O_2_Hb]_stim _using a paired t-test (Table 1) revealed statistical significance in the MI (p = 0.049) and IM (p < 0.001) conditions. No significant differences were detected between [HHb]_rest _and [HHb]_stim_. Figure 4 shows an example of a sample subject of the oxygenation changes from rest to stimulation period in each of the four conditions.

**Figure 5:**
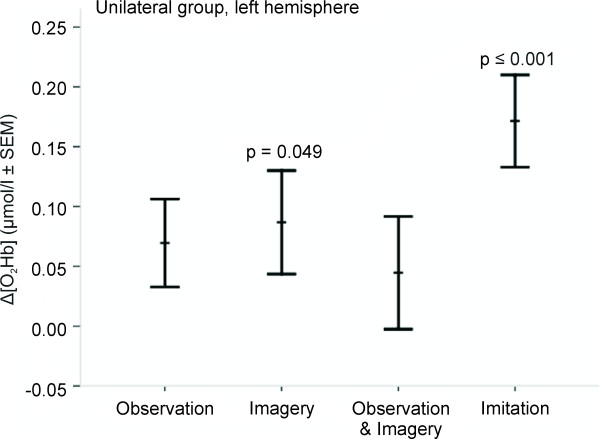
**Unilateral group recorded over left hemisphere: diagram of the Δ[O_2_Hb] amplitude changes with standard error of the mean (SEM) and statistical significances of paired t-test are shown**.

Inter-condition analysis of the mean amplitude changes of Δ[O_2_Hb] and Δ[HHb] between rest and stimulation periods between the four conditions using one-way ANOVA (Table 1, Figure 5) revealed neither a main effect of condition, nor statistical significant between the four conditions.

#### Bilateral group

In this group a smaller number of subjects was included, although sufficient to reach statistical significance.

In the left hemisphere, the mean Δ[O_2_Hb] (Table 2) were largest in condition IM_L, followed by IM_right, O_R, and O_L. Mean Δ[HHb] were largest in condition IM_L, followed by IM_R, O_L and O_R.

**Table 2:** Bilateral group.

Bilateral group [N = 8]	Observation right	Observation left	Imitation right	Imitation left
**Left hemisphere (μmol/l ± SD)**				

Mean Δ[O_2_Hb]	0.0924 ± 0.3369	0.0835 ± 0.4589	0.1905 ± 0.5515	0.2712 ± 0.4424

Mean Δ[HHb]	-0.0028 ± 0.1039	-0.0138 ± 0.1923	0.0206 ± 0.1569	0.0297 ± 0.1273

				

T-test, CI 95%				

[O_2_Hb] p = value	p = 0.016*	p = 0.046*	p = 0.003*	p < 0.001*

[HHb] p-value	p = 0.807	p = 0.523	p = 0.244	p = 0.040*

				

ANOVA, post-hoc-tests, Bonferroni 0.05		[HHb] p-value	[O_2_Hb] p = value	

	O - MI	p = 1.000	p = 1.000	

	O - O&MI	p = 1.000	p = 1.000	

	O - IM	p = 1.000	p = 0.080	

	MI - O&MI	p = 0.868	p = 0.822	

	MI - IM	p = 0.393	p = 0.056	

	O&MI - IM	p = 1.000	p = 1.000	

main effect on condition		p = 0.222	p = 0.028*	

				

**Right hemisphere (μmol/l ± SD)**				

Mean Δ[O_2_Hb]	0.1135 ± 0.3607	0.1091 ± 0.4261	0.2004 ± 0.4740	1.1475 ± 2.5449

Mean Δ[HHb]	0.0018 ± 0.1388	0.0037 ± 0.1441	0.0163 ± 0.1325	0.068 ± 0.1773

				

T-test, CI 95%				

[O_2_Hb] p = value	p = 0.006*	p = 0.025*	p < 0.001*	p = 0.001*

[HHb] p-value	p = 0.906	p = 0.817	p = 0.275	p = 0.001*

				

ANOVA, post-hoc-tests, Bonferroni 0.05		[HHb] p-value	[O_2_Hb] p = value	

	O - MI	p = 1.000	p = 1.000	

	O - O&MI	p = 1.000	p = 1.000	

	O - IM	p < 0.001*	p < 0.001*	

	MI - O&MI	p = 1.000	p = 1.000	

	MI - IM	p < 0.001*	p < 0.001*	

	O&MI - IM	p < 0.001*	p < 0.001*	

main effect on condition		p < 0.001*	p < 0.001*	

(Top) Mean signal amplitudes (μmol/l ± SD) of channels with significant concentration changes. Separate calculations for increases in [O2Hb], decreases in [HHb] in response to the four conditions for each group. Numbers were rounded to four decimal places. (Middle) Intra-condition statistical significance of the mean change between [O2Hb]rest and [O2Hb]stim and [HHb]rest and [HHb]stim using the paired t-test; confidence interval (CI) = 95%. (Bottom) Inter-condition statistical significance of mean changes of Δ[O2Hb] and Δ[HHb] between the four conditions using ANOVA. Shown are post-hoc tests (with Bonferroni correction); significant values (p ≤ 0.05) are highlighted by * (observation left = O_L, observation right = O_R, imitation left = IM_L, imitation right = IM_R)

On the right hemisphere, mean Δ[O_2_Hb] were largest in condition IM_L, followed by IM_R, O_R, and O_L. Mean Δ[HHb] were largest in condition IM_L, followed by IM_R, O_L and O_R. As also seen in the unilateral group a relatively high inter-subject variability was observed, as documented by the standard deviation (SD).

Intra-condition analysis (left hemisphere (LH), right hemisphere (RH)) of the median change between [O_2_Hb]_rest _and [O_2_Hb]_stim _using the paired t-test (Table 2) revealed statistical significant differences in conditions O_R (LH p = 0.016, RH p = 0.006), O_L (LH p = 0.046, RH p = 0.025), IM_R (LH p = 0.003, RH p < 0.001) and IM_L (LH p < 0.001, RH p = 0.001). Between [HHb]_rest _and [HHb]_stim _statistical significance was observed in condition IM_L (LH p = 0.040, RH p < 0.001).

**Figure 6:**
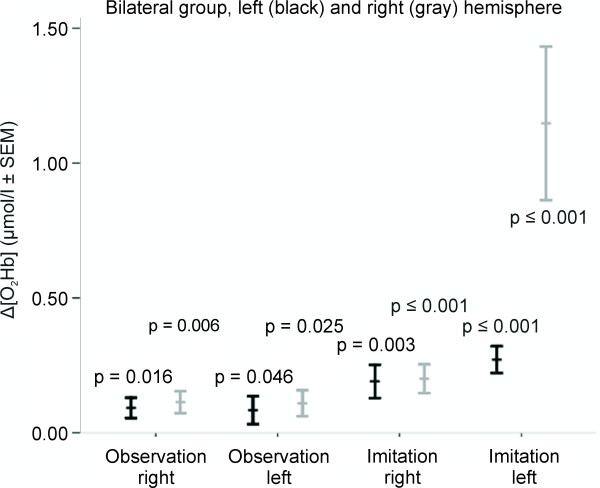
**Bilateral group recorded over left (black) and right (gray) hemisphere: diagram of the Δ[O_2_Hb] amplitude changes with standard error of the mean (SEM) and statistical significances of paired t-test are shown**.

Inter-condition analysis of the mean amplitude changes of Δ[O_2_Hb] and Δ[HHb] between the four conditions using one-way ANOVA (Table 2, Figure 6) revealed a main effect of condition for [O_2_Hb] (LH p = 0.028, RH p < 0.001) and for [HHb] (RH p < 0.001). Statistical significance was found for Δ[O_2_Hb] between-conditions O_R and IM_L (RH p < 0.001), O_L and IM_L (RH p = < 0.001) and IM_R and IM_L (RH p < 0.001); analog for Δ[HHb] between-conditions O_R and IM_L (RH p < 0.001), O_L and IM_L (RH p = < 0.001) and IM_R and IM_L (RH p < 0.001).

In the following discussion we concentrate on the observed [O_2_Hb] changes, since this parameter shows the relevant significant oxygenation changes, whereas [HHb] did show overall significant levels. This is supported by previous fNIRS work suggesting that interpretations about task-relevant activation increases are usually attributed to the prominent increases in [O_2_Hb] [40], whereas [HHb] is often not reported.

## Discussion

### Virtual reality based neurorehabilitation

Recent experimental evidence suggests that rapid advancement of VR technologies has great potential for the development of novel strategies for sensory-motor training in neurorehabilitation [41]. The combination with our wireless and portable fNIRS brain monitoring technique [12] is particularly appealing from a rehabilitation perspective as it allows therapists and patients unconstraint monitoring while testing and training motor performance [21,42].

In this study we provide evidence for the efficacy of our new VR neurorehabilitation system [9] by evaluating its effects on brain activation. In particular, we show that our VR system is able to elicit the action-observation system as described by the simulation hypothesis. Based on these results we aim in the long-term to develop a VR-fNIRS based BCI that providing the possibility of real-time neurofeedback combined with an assessment of training-induced cortical oxygenation changes.

### Observation, imagery and imitation

From the comparisons between stimulation and rest periods, our results confirm the simulation hypothesis in accordance with well-known findings in fMRI and EEG [3,14,15,43,44] and previous fNIRS studies [21-25,45] that have shown that oxygenation changes can be found within the same secondary motor areas during observation, motor imagery and overt motor execution (unilateral and bilateral group, Figure 5 and 6). Although not all of the observed changes reached statistical significance, our results revealed that averaged Δ[O_2_Hb] during observation and motor imagery were approximately one-third lower compared to the imitation task. This result is in line with the previous studies mentioned above where both imagery and observation have been reported to elicit consistently lower oxygenation changes.

### Inter-subject variability

We observed a high inter-subject variability in Δ[O_2_Hb] in both our samples. General reasons for variability between individuals may be effects of anatomical variance such as thickness of the skull or cerebrospinal fluid layers [46,47]. Another contributing factor might be that our subjects had no prior specific experience in the tasks presented. They were not specifically trained to perform the tasks prior to the experiment (but only received a short practice trial), yet this has been done in a previous fNIRS controlled BCI [24]. Therefore, in our untrained subjects, inter-subject variability in the hemodynamic response patterns might have been higher than it would have been after substantial pre-experimental training. The question of the extent to which a person is able to generate a mental representation of movements is even more relevant in the assessment of individuals following brain injury. Lesions involving specific cortical areas may impair certain imagery abilities [48], such as overall slowing of imagery processes resulting in modified temporal characteristics of motor imagery [49,50].

### Bilateral oxygenation

As observed in previous studies, brain activation in response to executed or imagined actions can differ depending on the hemisphere recorded [51-53]. In general, unimanual tasks show hemispheric asymmetry with predominant activation of the contralateral hemisphere controlling the moving hand, as assessed by fMRI and PET [30-33]. Additionally, ipsilateral activation is both found in M1 and shifted laterally, ventrally, and anteriorly towards PMC for unimanual tasks with respect to that observed during contralateral hand movements [54-60]. Accordingly, we observed ipsi- and contralateral oxygenation changes, both during observation and imitation.

The difference observed between the unilateral and the bilateral group is concerned about the aspect of handedness. Interestingly, we found that performance during the condition IM_L (imitation with the subject's left non-dominant hand) revealed larger Δ[O_2_Hb] in both hemispheres as compared to IM_R (imitation with the subject's right dominant hand) (Figure 6). Further, the Δ[O_2_Hb] in the right hemisphere during movement of the subjects' left hand (i.e. the non-dominant, contralateral hand) is considerably larger than that in the left hemisphere during ipsilateral movement. Additionally, in the left hemisphere during ipsilateral movement (non-dominant hand) the Δ[O_2_Hb] was larger than that observed during contralateral movement (dominant hand; according to the unilateral group). Figure 5 and 6 reflect these findings showing the observed inter-condition differences in the right hemisphere including lower level Δ[O_2_Hb] amplitude during observation as compared to imitation (Figure 6). These findings might be explained by the hand dominance of our right-handed sample. Previous fMRI studies described that non-dominant hand movements appear to require more cortical activity and therefore may result in greater recruitment of ipsi- and contralateral cortical motor areas [61], perhaps because they are less 'automatic'. It has been further observed that this ipsilateral activation was most pronounced in pre-central areas (presumably corresponding to secondary motor areas) during both dominant and non-dominant performance [62]. However, further fNIRS studies are needed to confirm whether or not our findings of larger Δ[O_2_Hb] during non-dominant performance are in fact caused by the right-handedness of our sample.

### Neurorehabilitative potential of combined VR NIRS applications

Taken together the findings of the uni- and bilateral groups, the results show that our VR system can activate the action-observation system as described by the simulation hypothesis. In particular, 1) the study provides evidence that fNIRS recording does not impede interaction with the VR environment This point is an important precondition for further development of combined VR-fNIRS based applications for use in neurorehabilitation. It increases usability in that it requires a short time to fit fNIRS sensor important for therapy. Further, the results revealed two factors that need to be taken into account when dealing with fNIRS signals aimed to provide a basis for neural interfaces: 2) The inter-subject variability is obvious at the group level and will be even more prominent at he single subject level. The reasons for inter-subject variability, i.e. individual experience in motor imagery performance, physiological and anatomical differences, require further assessment. 3) The combined factors of recording side, i.e. uni- or bilateral hemispheres, as well as hand side, i.e. left or right hand used during motor or imagery tasks, need to be taken into account. Our findings may reflect an aspect of handedness in right-handed subjects who may require more cortical activity when using the non-dominant hand. Future studies may include both left-handers and right-handers. Considering these factors may contribute to differentiation of individual oxygenation pattern and permit classification of activation tasks used for neurofeedback or BCI applications.

### Study limitations

Although the present study revealed interesting results concerning the potential of the new wireless NIRS system, it was subject to some known limitations. We did not record an electromyogram (EMG) in order to exclude the presence of muscular activation during observation and motor imagery. It could be claimed that weak motor activity might have been present during the imagery tasks. However, previous neuroimaging studies suggested that brain signals during imagery of hand motor tasks are not correlated with EMG activity [63]. Another possible limitation is that we referenced the positioning of the NIRS device according to the 10-20 system [35]. However, this positioning may be inaccurate due to inter-subject variability in anatomical head size and shape, and the location on underlying (pre-)motor areas. The location of NIRS recording can therefore generally only be assumed to have correctly covered the preferred areas, i.e. in our case secondary motor areas.

## Conclusion

This study shows that our combined VR-fNIRS based neurorehabilitation system is able to activate the action-observation system as described by the simulation hypothesis during performance of observation, motor imagery and imitation of hand actions elicited by a VR environment. Further, in accordance with previous studies, the findings of this study revealed that both inter-subject variability as well as handedness needs to be taken into account when recording in untrained subjects. In the long term, these findings are of relevance for the VR-fNIRS instrument's potential in neurofeedback applications.

LH conceived of the study, conducted the fNIRS recordings, carried out the statistical analysis, and drafted the manuscript. TM and FS carried out the MATLAB^® ^pre-processing. KE and DK participated in the design of the study. MW participated in the design and coordination of the study. All authors read and approved the final manuscript.

## Declaration of competing interests

The authors declare that they have no competing interests.
