# Correction: Testing the potential of a virtual reality neurorehabilitation system during performance of observation, imagery and imitation of motor actions recorded by wireless functional near-infrared spectroscopy (fNIRS)

**DOI:** 10.1186/1743-0003-10-16

**Published:** 2013-02-08

**Authors:** Lisa Holper, Thomas Muehlemann, Felix Scholkmann, Kynan Eng, Daniel Kiper, Martin Wolf

**Affiliations:** 1 Biomedical Optics Research Laboratory (BORL), Division of Neonatology, Department of Obstetrics and Gynecology, University Hospital Zurich, Frauenklinikstrasse 10, 8091, Zurich, Switzerland; 2 Institute of Neuroinformatics (INI), University of Zurich and ETH Zurich, Winterthurerstrasse 190, 8057, Zurich, Switzerland; 3 Molecular Imaging and Functional Pharmacology, Institute for Biomedical Engineering, ETH and University of Zurich, Wolfgang-Pauli-Strasse 27, 8093, Zurich, Switzerland

## Correction

Following publication of our article [1], we realised that some of the statistical tests used were not appropriate. We have now conducted the appropriate statistical tests, and updated the relevant tables, figures five and six (Figures 1 and 2 here, respectively) and conclusions accordingly.

**Figure 1:**
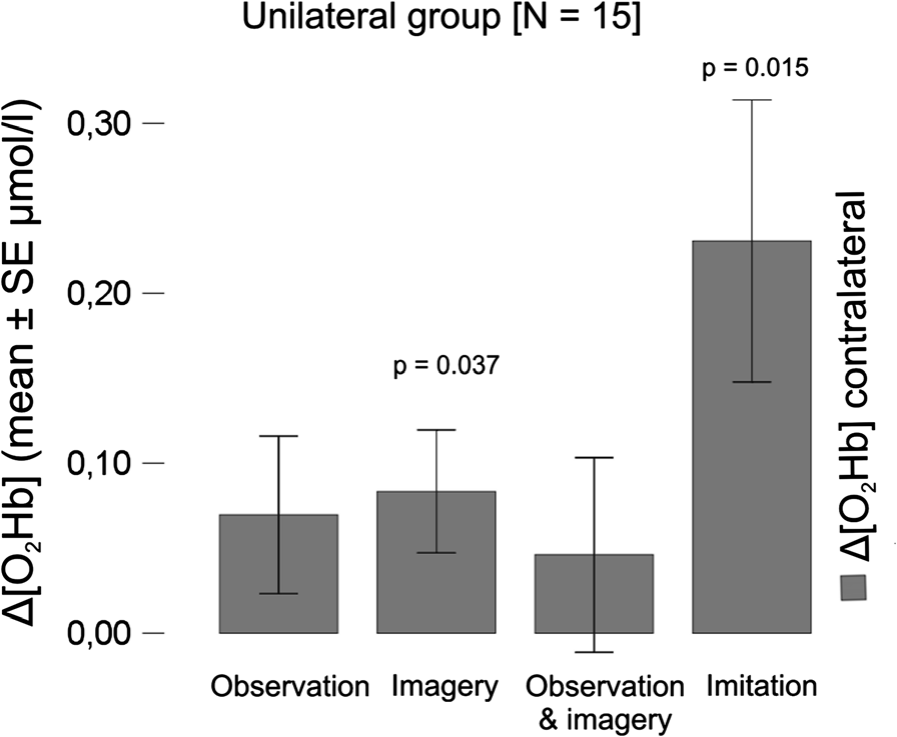
**Unilateral group recorded over contralateral hemisphere: shown are the Δ[O**_**2**_**Hb] amplitude changes with standard error of the mean (SEM) and statistical significances of repeated measures ANOVA.**

**Figure 2:**
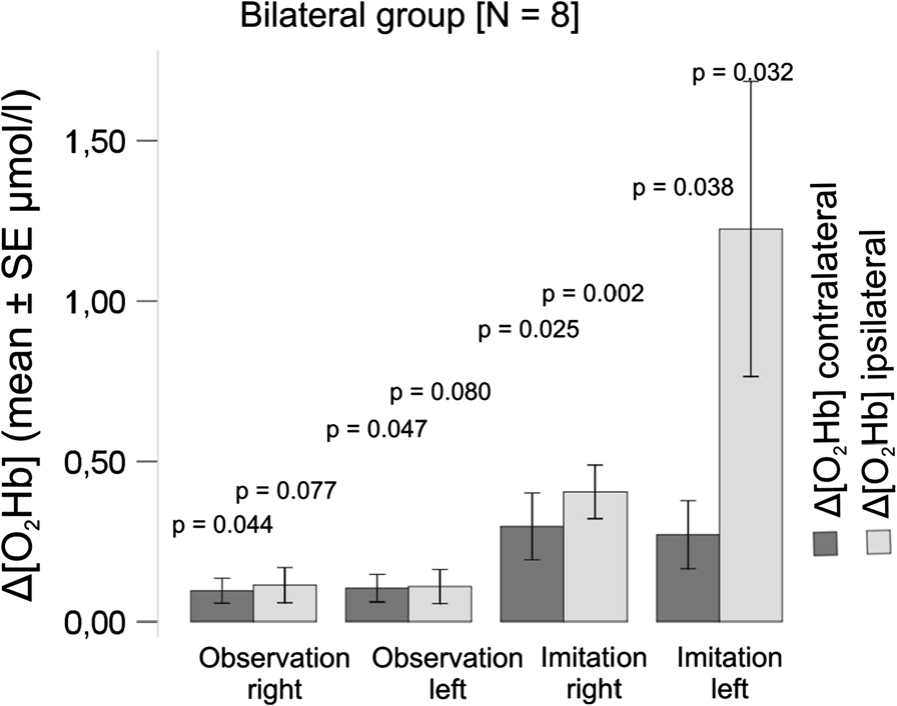
**Bilateral group recorded over contra- (dark gray) and ipsilateral (light gray) hemisphere: shown are the Δ[O**_**2**_**Hb] amplitude changes with standard error of the mean (SEM) and statistical significances of repeated measures ANOVA.**

For both the unilateral and the bilateral groups, analyses were recalculated.

Intra-condition differences:

• original publication [1]: paired *t*-test using means per trial for unilateral and bilateral group

• update: one-way repeated measures ANOVA using means per subject for unilateral and bilateral group

Inter-condition differences:

• original publication [1]: one-way ANOVA using means per trial for unilateral and bilateral group

• update: one-way repeated measures ANOVA using means per subject for unilateral and bilateral group

## Conclusions

For the unilateral group (Table 1 and Figure 1), no changes in significance levels were found in Δ[O_2_Hb] signals. For the bilateral group (Table 2 and Figure 2), the main differences compared to the original publication are that the intra-condition differences ([O_2_Hb]_rest_ versus [O_2_Hb]_stim_) for the two conditions ‘Observation Right’ (O_R, p = 0.077) and ‘Observation Left’ (O_L, p = 0.080) recorded over the ipsilateral hemisphere do not reach significant level any more. Hence, the paragraphs discussing the intra-condition significances in those two conditions (sections *Observation, imagery and imitation* and *Bilateral oxygenation* of the *Discussion*) are only applicable for the contralateral hemisphere. Further, in both groups changes in significance levels were found for Δ[HHb]. However, since the *Discussion* and *Conclusion* of the originally published article only focuses on the concentration changes found in Δ[O_2_Hb], this aspect does not change these sections.

**Table 1:** Unilateral group

**Unilateral group [N = 15]**	**Observation**	**Motor imagery**	**Observation &motor imagery**	**Imitation**
**Left hemisphere (contralateral) (μmol/l ± SD)**				
Mean ∆ [O_2_Hb]	0.06953 ± 0.1800	0.0833 ± 0.1404	0.0460 ± 0.2218	0.2309 ± 0.3212
Mean ∆ [HHb]	−0.0051 ± 0.03855	0.0356 ± 0.0771	−0.0089 ± 0.0963	0.0079 ± 0.0832
Intra-condition, ANOVA, repeated measures				
[O_2_Hb] rest-stim	p = 0.157	p = 0.037*	p = 0.435	p = 0.015*
[HHb] rest-stim	p = 0.612	p = 0.097	p = 0.727	p = 0.717
Inter-condition ANOVA, repeated measures, post-hoc-tests, Bonferroni 0.05		**∆ [HHb]**	**∆ [O**_**2**_**Hb]**	
	O – MI	p = 0.347	p = 1.000	
	O – O&MI	p = 1.000	p = 1.000	
	O – IM	p = 1.000	p = 0.286	
	MI – O&MI	p = 0.132	p = 1.000	
	MI – IM	p = 1.000	p = 0.622	
	O&MI – IM	p = 1.000	p = 0.321	
Main effect on condition		p = 0.253	p = 0.062	

(Top) Mean signal amplitudes (μmol/l ± SD) of channels with significant concentration changes. Separate calculations for increases in [O_2_Hb], decreases in [HHb] in response to the four conditions for each group. Numbers were rounded to four decimal places. (Middle) Intra-condition statistical significance of the mean changes between [O_2_Hb]_rest_ and [O_2_Hb]_stim_ and [HHb]_rest_ and [HHb]_stim_ repeated measures ANOVA. (Bottom) Inter-condition statistical significance of mean changes of ∆ [O_2_Hb] and ∆ [HHb] between the four conditions using repeated measures ANOVA. Shown are post-hoc tests (with Bonferroni correction); significant values (p ≤ 0.05) are highlighted by * (observation = O, motor imagery = MI, observation & motor imagery = O & MI, imitation = IM).

**Table 2:** Bilateral group

**Bilateral group [N = 8]**	**Observation right**	**Observation left**	**Imitation right**	**Imitation left**
**Left hemisphere (contralateral) (μmol/l ± SD)**				
Mean ∆ [O_2_Hb]	0.1231 ± 0.1506	0.1231 ± 0.1507	0.3941 ± 0.4598	0.3715 ± 0.4289
Mean ∆ [HHb]	−0.0056 ± 0.0676	−0.0408 ± 0.0915	0.0371 ± 0.1131	0.0474 ± 0.0665
Intra-condition, ANOVA, repeated measures				
[O_2_Hb] rest-stim	p = 0.044*	p = 0.047*	p = 0.025*	p = 0.038*
[HHb] rest-stim	p = 0.821	p = 0.247	p = 0.384	p = 0.084
Inter-condition, ANOVA, repeated measures, post-hoc-tests, Bonferroni 0.05		**∆ [HHb]**	**∆ [O**_**2**_**Hb]**	
	O_R – O_L	p = 1.000	p = 1.000	
	O_R – IM_R	p = 1.000	p = 0.519	
	O_R – IM_L	p = 1.000	p = 0.862	
	OL_ – IM_R	p = 0.227	p = 0.486	
	O_L – IM_L	p = 0.223	p = 0.777	
	IM_R – IM_L	p = 1.000	p = 1.000	
Main effect on condition		p = 0.072	p = 0.119	
**Right hemisphere (ipsilateral) (μmol/l ± SD)**				
Mean ∆ [O_2_Hb]	0.1541 ± 0.0735	0.1957 ± 0.1957	0.4036 ± 0.2097	1.3728 ± 1.6143
Mean ∆ [HHb]	−0.0113 ± 0.0334	0.0068 ± 0.0274	0.0235 ± 0.0402	0.7016 ± 1.9167
Intra-condition, ANOVA, repeated measures				
[O_2_Hb] rest-stim	p = 0.077	p = 0.080	p = 0.002*	p = 0.032*
[HHb] rest-stim	p = 0.367	p = 0.502	p = 0.142	p = 0.335
Inter-condition, ANOVA, repeated measures, post-hoc-tests, Bonferroni 0.05		**∆** [**HHb**]	**∆** [**O**_**2**_**Hb**]	
	O_R – O_L	p = 1.000	p = 1.000	
	O_R – IM_R	p = 0.445	p = 0.014*	
	O_R – IM_L	p = 1.000	p = 0.324	
	OL_ – IM_R	p = 1.000	p = 0.015*	
	O_L – IM_L	p = 1.000	p = 0.231	
	IM_R – IM_L	p = 1.000	p = 0.710	
Main effect on condition		p = 0.384	p = 0.008*	

(Top) Mean signal amplitudes (μmol/l ± SD) of channels with significant concentration changes. Separate calculations for increases in [O_2_Hb], decreases in [HHb] in response to the four conditions for each group. Numbers were rounded to four decimal places. (Middle) Intra-condition statistical significance of the mean change between [O_2_Hb]_rest_ and [O_2_Hb]_stim_ and [HHb]_rest_ and [HHb]_stim_ using repeated measures ANOVA. (Bottom) Inter-condition statistical significance of mean changes of ∆ [O_2_Hb] and ∆ [HHb] between the four conditions using repeated measures ANOVA. Shown are post-hoc tests (with Bonferroni correction); significant values (p ≤ 0.05) are highlighted by * (observation left = O_L, observation right = O_R, imitation left = IM_L, imitation right = IM_R).
